# Olive oil nanoemulsion containing curcumin: antimicrobial agent against multidrug-resistant bacteria

**DOI:** 10.1007/s00253-024-13057-x

**Published:** 2024-02-27

**Authors:** Maine Virgínia Alves Confessor, Maria Anndressa Alves Agreles, Luís André de Almeida Campos, Azael Francisco Silva Neto, Joyce Cordeiro Borges, Rodrigo Molina Martins, Alexsandra Maria Lima Scavuzzi, Ana Catarina Souza Lopes, Elisangela Afonso de Moura Kretzschmar, Isabella Macário Ferro Cavalcanti

**Affiliations:** 1 Keizo Asami Institute (iLIKA), Federal University of Pernambuco (UFPE), Prof. Moraes Rego Avenue, 1235, Cidade Universitária, CEP, Recife, Pernambuco, 50670-901 Brazil; 2 University Center UNIFACISA, Manoel Cardoso Palhano, 124-152, Itararé, CEP, Campina Grande, Paraiba 58408-326 Brazil; 3 Department of Tropical Medicine, Federal University of Pernambuco (UFPE), Recife, Pernambuco Brazil; 4 Department of Biotechnology, Federal University of Paraíba (UFPB), João Pessoa, Paraíba, Brazil; 5 Laboratory of Microbiology and Immunology, Academic Center of Vitória (CAV), Federal University of Pernambuco (UFPE), Vitória de Santo Antão, Pernambuco, Brazil

**Keywords:** Olive oil, *Curcuma longa*, Nanotechnology, Infection, Bacterial resistance

## Abstract

**Abstract:**

The present work aimed to develop, characterize, and evaluate the antibacterial and antibiofilm activity of two nanoemulsions (NEs) containing 500 µg/mL of curcumin from *Curcuma longa* (CUR). These NEs, produced with heating, contain olive oil (5%) and the surfactants tween 80 (5%) and span 80 (2.5%), water q.s. 100 mL, and were stable for 120 days. NE-2-CUR presented *Ø* of 165.40 ± 2.56 nm, *PDI* of 0.254, *ζ* of − 33.20 ± 1.35 mV, pH of 6.49, and Entrapment Drug Efficiency (*EE*) of 99%. The NE-4-CUR showed a *Ø* of 105.70 ± 4.13 nm, *PDI* of 0.459, *ζ* of − 32.10 ± 1.45 mV, pH of 6.40 and *EE* of 99.29%. Structural characterization was performed using DRX and FTIR, thermal characterization using DSC and TG, and morphological characterization using SEM, suggesting that there is no significant change in the CUR present in the NEs and that they remain stable. The MIC was performed by the broth microdilution method for nine gram-positive and gram-negative bacteria, as well as *Klebsiella pneumoniae* clinical isolates resistant to antibiotics and biofilm and efflux pump producers. The NEs mostly showed a bacteriostatic profile. The MIC varied between 125 and 250 µg/mL. The most sensitive bacteria were *Staphylococcus aureus* and *Enterococcus faecalis*, for which NE-2-CUR showed a MIC of 125 µg/mL. The NEs and ceftazidime (CAZ) interaction was also evaluated against the *K. pneumoniae* resistant clinical isolates using the Checkerboard method. NE-2-CUR and NE-4-CUR showed a synergistic or additive profile; there was a reduction in CAZ MICs between 256 times (K26-A2) and 2 times (K29-A2). Furthermore, the NEs inhibited these isolates biofilms formation. The NEs showed a MBIC ranging from 15.625 to 250 µg/mL. Thus, the NEs showed physicochemical characteristics suitable for future clinical trials, enhancing the CAZ antibacterial and antibiofilm activity, thus becoming a promising strategy for the treatment of bacterial infections caused by multidrug-resistant *K. pneumoniae*.

**Key points:**

• *The NEs showed physicochemical characteristics suitable for future clinical trials.*

• *The NEs showed a synergistic/additive profile, when associated with ceftazidime.*

• *The NEs inhibited biofilm formation of clinical isolates.*

**Supplementary Information:**

The online version contains supplementary material available at 10.1007/s00253-024-13057-x.

## Introduction

The pathogens microorganisms’ resistance to drugs is a current problem and has direct implications for patient safety, resulting in hospital admissions, prolonging the patient’s stay, increasing treatment time, and the risk of death (Nandhini et al. 2022; Reddy et al. 2020). Furthermore, according to the World Health Organization (WHO), at least 700,000 people die every day from diseases caused by bacteria that are resistant to antibiotics. In 2019, almost 3 million cases of antibiotic-resistant infections were reported in the USA (WHO - World Health Organization 2017, 2019), evidencing the strong demand in the search for new alternatives to multidrug-resistant infections.

Considering bacterial resistance as an epidemic with serious consequences, antimicrobials of natural origin are effective and, in most cases, economical alternatives. *Curcuma longa* L, popularly known as curcumin, turmeric, or saffron, is a plant belonging to the Zingiberaceae family, which is popularly sold as a yellowish powder, mainly from the rhizomes. It is a product widely used in cooking because it is a strong colorant and because of its medicinal activities (Liakopoulou et al. 2021; Martins et al. 2013; Zamarioli et al. 2015).

Turmeric has several active compounds, with an emphasis on curcuminoids, which include curcumin (77% of curcuminoids), demethoxycurcumin (18%), and bisdemethoxycurcumin (5%), among them, the most important is curcumin, which corresponds to about 3–4% of the plant (Aly et al. 2017). This phytoconstituent has several therapeutic effects, such as, anti-inflammatory, antitumor, and antimicrobial action for several microorganisms, among them, gram-negative and gram-positive bacteria (Alves et al. 2017; Karimi et al. 2018; Reddy et al. 2020). Furthermore, its potential against various types of viruses has been recognized, such as HPV-16, the various dengue viruses and HIV, and its use has been validated, including against vulvar intraepithelial neoplasia (VIN), considering that its use resulted in the death of HPV-infected cells when associated with photodynamic therapy (Bonfim et al. 2020; Mirani et al. 2019; Nabila et al. 2020, 2021).

Curcumin has its maximum absorption at a wavelength of 430 nm, boiling point of 183 °C, low solubility in water, low bioavailability, and reduced chemical stability, being sensitive to various conditions. Due to its low water solubility and susceptibility to factors such as pH, temperature, oxidation, and light, curcumin has limitations in its use (Liakopoulou et al. 2021; Zamarioli et al. 2015). It also undergoes extensive first-pass metabolism in the body due to glucuronidation and sulphation in its molecule, which produce derivatives with significantly lower biological activity, and which are more easily eliminated (Vollono et al. 2019).

Thus, despite its broad potential as a medicinal resource, it is recognized that turmeric has characteristics that compromise or hinder its use in the food and pharmaceutical industry (Aljabri et al. 2022; Martins et al. 2013). In this sense, mechanisms that favor the efficiency of the delivery of these phytochemicals are of paramount importance, considering that absorption is essential for their action.

In this way, nanotechnology and its various systems are strategies for the use of these bioactives, since, after being nanoencapsulated, they result in better hydrophilicity, chemical stability, sustained release, and adequate characteristics for the industry when compared to their free form (Azami et al. 2018; Liakopoulou et al. 2021).

In this scenario, nanoemulsions (NEs) play an important role. These are colloidal systems and, like conventional emulsions, are formed by two immiscible phases, one aqueous and the other oily, stabilized by a surfactant, forming droplets on a nanometric scale, which can encapsulate, protect, and release bioactive components. NEs are, therefore, one of the systems that can be used as carriers or delivery systems for lipophilic compounds, nutraceuticals, such as curcumin, or drugs, flavorings, antioxidants, and antimicrobial agents, which have been increasingly used in the food industry or pharmaceutical (Aljabri et al. 2022).

In this sense, it is known that the olive oil (*Olea europaea* L.) has been widely used due to its properties and multiple uses since antiquity. It is a monounsaturated oil, which contains high levels of vitamin E, carotenoids, and polyphenols, also presenting phytosterols, pigments, and hydrocarbons (Conte et al. 2018). Olive oil contains a high concentration of oleic acid in its composition, ranging from 56 to 84%; there are also essential polyunsaturated fatty acids, such as linoleic acid (3.5 to 21%) and linolenic acid (< 1 to 5%).

Phenolic compounds are also present in this food and stand out for having chemopreventive characteristics, since these compounds can act to inhibit platelet aggregation, contributing to the transcription of the mRNA of the antioxidant enzyme glutathione peroxidase. There are also oleacein, oleocanthal, apigenin, and luteolin, substances that also have great antioxidant activity (Lanza and Ninfali 2020).

Thus, the olive oil also has a protective effect and is, therefore, considered an oil rich in medicinal properties, easy to acquire, and at an acceptable cost (UNIRIO 2020). Therefore, this work aimed to develop, characterize, and evaluate the antibacterial and antibiofilm activity of curcumin from *Curcuma longa* L. (CUR) in olive oil nanoemulsions.

## Material and methods

### Material

Culture media reagents (Müeller Hinton Broth—MHB; Müeller Hinton agar—MHA), ceftazidime (CAZ), curcumin from *Curcuma longa* L. (CUR), and surfactants (Tween 80 and Span 80) were obtained from Himedia® and Sigma-Aldrich®. All solvents (Dimetilsulfoxide—DMSO, saline solutions) were purchased from Merck®^.^

The products are as the following:

Ceftazidime: Formula—C22H22N6O7S2, Molecular weight—636.65 g/mol, CAS number—78,439–06-2, concentration—100%). Curcumin: Formula—C21H20O6, Molecular weight—368.38 g/mol, CAS number—458–37-7, Purity (HPLC)— ≥ 65%.

### Experimental design of olive oil nanoemulsions containing curcumin from *C. longa*

Initially, a pre-formulation study was carried out to obtain the NEs, evaluating the size and distribution of the particles, the effects of the emulsification method (spontaneous emulsification vs. high-intensity agitation), hydrophilic-lipophilic balance (HLB), type and concentration of surfactant, and type of oil used.

NEs were prepared by the spontaneous emulsification method adapted from McClements (2011). The formulations were prepared from the observation of the HLB values of the surfactants to obtain NEs with hydrophilic-lipophilic characteristics suitable for their clinical application in the treatment of infectious diseases in humans.

Briefly, the oil phase was stirred at 300 rpm for 10 min. Then, water was added using a syringe and stirred for 30 min. Then, the emulsions were taken to the Q55 Sonicator ultrasonic processor (Qsonica Sonicators, Newtown, Connecticut) at 50° amplitude for 10, 20, or 30 min for formulas NE-1 and NE-2, NE-3 and NE-4, and NE-5 and NE-6, respectively. The NE-1, NE-3, and NE-5 formulas were prepared at room temperature (~ 25 °C), while the others were heated to approximately 40 °C throughout the process. Subsequently, the NEs were analyzed for their physicochemical properties, including particle size (*Ø*), polydispersion index (*PDI*), zeta potential (*ζ*), pH, and stability study.

To encapsulate the curcumin from *Curcuma longa* L. (CUR), NE-2 and NE-4 formulas were selected, therefore obtaining the following NEs: (1) NE-2-CUR—Nanoemulsion 2 containing curcumin and (2) NE-4-CUR—Nanoemulsion 4 containing curcumin. To prepare the NEs, after stirring the oil phase, CUR (2000, 1000, and 500 µg/mL) was added, and the stirring process was repeated for 10 min. Afterward, the formulation process was performed, as described for the NEs without curcumin.

The entire formulation phase was performed at the Federal University of Paraíba Laboratory (LANNI/UFPB) and in independent triplicates.

### Physical–chemical evaluation of nanoemulsions

#### Hydrogen potential analysis (pH), color, and macroscopic morphological appearance

The pH of the NEs was carried out at room temperature (25 °C), using a pH meter (MS TECNOPON LUCA-210), calibrated with buffer solutions of pHs 4.0 and 7.0. Measurements were made immediately after preparing the formulations and after 30 days.

Macroscopically, the observation of the color variation and morphological aspect between the formulations was carried out in terms of whether they were opaque or translucent and the possible changes throughout the experiment.

#### Characterization of nanoemulsions

Samples of the formulations were submitted for analysis using the photon correlation spectroscopy technique, also known as dynamic light scattering (DLS). The *Ø*, *PDI*, and *ζ* are fundamental parameters to determine the stability and potential of the formulations for application in in vivo and clinical tests. The mean diameter and *PDI* values, an indication of the variance in the size distribution, were obtained for NE samples using the Zetasizer Nano ZS equipment (Malvern Panalytical, UK), with a dispersion angle fixed at 90° and at 25 °C. For particle size analysis (*Ø*), 50 µL of nanoemulsions was diluted in 950 µL of ultrapure water (Milli Q®, Millipore, USA). The results were analyzed using the Zetasizer 7.13 program (Malvern Panalytical, UK). The surface charge of the nanoparticles was established by determining the Zeta Potential (*ζ*). To measure *ζ*, 50 µL of the samples was diluted in 950 µL of purified water using the Zetasizer. The results were obtained by averaging the reading of triplicates.

Samples were analyzed for determining the curcumin content at 420 nm using a curcumin standard curve with concentrations ranging from 0.5 to 6 μg/mL. Absorbances were obtained by UV/vis spectrophotometry (UV-1900 UV–Vis-Shimadzu, Kyoto, Japan). The determination of the Entrapment Drug Efficiency (*EE*) was performed using the ultrafiltration-centrifugation technique. Samples (400 µL) were centrifuged at 9000 rpm for 1 h min at 25 °C. The supernatant was collected and added to 1 mL of methanol, being measured by a spectrophotometer at 420 nm. The CUR *EE* was calculated by Eq. (1), based on the straight line equation obtained by the standard CUR curve:

1 $$\mathrm{\% }EE=\frac{\mathrm{Curcumin\;content }-\mathrm{ Unloaded\;curcumin}}{\mathrm{Curcumin\;content}}$$

Data are presented as the percentage of the initial drug entrapment in NEs. The determination of *EE* was performed at the Multiuser Laboratory of Pharmaceutical Sciences (LAMCIF)—UNIFACISA and in independent triplicates.

Also, the obtained formulations were evaluated regarding the stability test that aims to establish the durability of the preparations. In this test, the condition of the systems was evaluated right after manufacture and at regular time intervals (7, 15, 30, 60, 90, 120 days).

### Scanning electron microscopy of nanoparticles

For morphological analysis of the nanoparticles, the scanning electron microscopy (SEM) technique was used at a magnification of 40 × and 100 × (model CX31, Olympus, Japan). NEs containing CUR were diluted in ultrapure water at a ratio of 1:1 v/v NEs:water. The NEs were spread on glass plates and kept at room temperature until complete drying. After drying, the plates were coated with gold/palladium under an argon atmosphere (FINE COAT, ION SPUTTER JFC-1100) and observed under a ZEISS scanning electron microscope at 25 kV, model EVO-LS15 (da Rosa et al. 2015).

### Differential scanning calorimetry (DSC) and thermogravimetry (TG)

DSC analysis was performed using a DSC Q10 (V9.9 Build 303, USA). Each sample (2 mg) was placed in closed aluminum containers. Subsequently, the samples were subjected to heating from 20 to 200 °C in an atmosphere of nitrogen gas at 60 mL/min. An aluminum container was taken as a reference.

For the TG analysis, a Q500 TGA apparatus (V20.13 Build 39, USA) was used. Samples were weighed with a total of 100 mg. All experiments were conducted under a nitrogen atmosphere with a feed flow of 15 mL/min. The samples were heated from 20 to 600 °C at 10 °C/min.

### X-ray diffraction analysis

The structural properties of the NEs were characterized using an XRD 7000 diffractometer (Shimadzu, Kyoto, Japan) to obtain the data, with an open angle of 2θ in the range of 5–70°, θ–θ system, using Cu radiation (*λ* = 1 0.5418 Å), in a step of 0.02° (θ), and 1.0 s of interval for each sample.

### Fourier transform infrared spectroscopy

This analysis was performed using a Spectrum TM 400 FT-IR/FTNIR spectrometer (Perkin Elmer, Waltham, MA, USA) processed on a KBr wafer with a resolution of 4 cm^−1^, scanning speed of 0.2 cm^−1^ of 4000 at 500 cm^−1^ for the NEs produced.

## Evaluation of antibacterial activity

All experiments were carried out at the Clinical Microbiology Laboratory of the Keizo Asami Institute of the Federal University of Pernambuco (iLIKA/UFPE) and in independent triplicates.

## Determination of the minimum inhibitory concentration (MIC)

The bacteria used in this study were *Acinetobacter baumannii* ATCC 19606, *Escherichia coli* ATCC 13846, *E. coli* ATCC 25922, *Enterococcus faecalis* ATCC 29212, *Klebsiella pneumoniae* ATCC 13883, *K. pneumoniae* ATCC 700603, *Pseudomonas aeruginosa* ATCC 27853, *Staphylococcus aureus* ATCC 25923, *S. aureus* ATCC 33591, and the clinical isolates of *K. pneumoniae* resistant to antibiotics and biofilm and efflux pump producers K25-A2, K26-A2, K29-A2, and K31-A2.

Antibacterial activity was performed by determining the minimum inhibitory concentration (MIC) using the broth microdilution method according to the *Clinical and Laboratory Standards Institute* (CLSI 2020). Initially, Müeller Hinton Broth (MHB) was added to microdilution plates. Then, the NEs were added by serial dilution to obtain the concentration range from 0.5 to 250 µg/mL. The bacterial suspensions were adjusted to a density of 0.5 on the McFarland scale and then deposited in the wells, obtaining a final concentration of 10^5^ CFU/well. Subsequently, the plates were incubated at 35 ± 2 °C for 24 h, and after incubation, the reading was performed by spectrophotometry at a wavelength of 620 nm (Multiskan FC, Thermo Scientific, Madrid, Spain). The MIC was determined as the lowest concentration capable of inhibiting more than 80% of microbial growth.

The minimum bactericidal concentration (MBC) was determined after the MIC results. An aliquot of the sample from the wells where there was no growth was inoculated in Müeller Hinton agar (MHA) and the plates were incubated at 35 ± 2 °C for 24 h. After this period, MBC was determined as the lowest concentration at which there is no microbial growth (CLSI 2020).

### Evaluation of the interaction of nanoemulsions with ceftazidime against resistant clinical isolates of *Klebsiella pneumoniae*

The bacteria used in this study were *Klebsiella pneumoniae* ATCC 13883 and the clinical isolates of *K. pneumoniae* K25-A2, K26-A2, K29-A2, and K31-A2, which are biofilm and efflux pump producers*.*

To evaluate the interaction between NEs containing CUR and ceftazidime (CAZ) against CAZ-resistant strains, the *Checkerboard* method was performed (Sopirala et al. 2010). Initially, 95 μL of culture medium was added to each well of 96-well plates. To obtain the final MIC and dilutions with lower values than the MIC of the respective compounds, CAZ was added on the X-axis and NEs on the Y-axis. Finally, the bacterial concentration was adjusted to a density of 0.5 on the McFarland scale to obtain the final concentration of 10^5^ CFU/well. Wells in column 11 were used as growth controls, containing only the culture medium and inoculum, while wells in column 12 were used as sterile controls, containing only the culture medium. The microplates were incubated at 35 ± 2 °C for 24 h, and after this period, the reading was performed at 620 nm. MIC was determined as the lowest concentration capable of inhibiting more than 80% of microbial growth.

To classify the interaction between the compounds, the Fractional Inhibitory Concentration Index (FICI) was calculated according to the equation ∑FICI = FICIa + FICIb = (MICa + b/MICa) + (MICa + a/MICb), where MICa + b and MICb + a are the MICs of the compounds in the combination where interaction was found and MICa and MICb are the MICs of the compounds alone. Results were interpreted as follows: (1) synergism—FICI ≤ 0.5; (2) additive—0.5 > FICI ≤ 1.0; (3) indifferent—1 > FICI ≤ 4.0; (4) antagonism—FICI > 4.

### Evaluation of antibiofilm activity against resistant clinical isolates of *Klebsiella pneumoniae*

The bacteria used in this study were the clinical isolates of *K. pneumoniae* K25-A2, K26-A2, K29-A2, and K31-A2, which are efflux pump and biofilm producers. The isolates were collected in hospitals in Recife-PE, Brazil. The *K. pneumoniae* strains came from the study by Scavuzzi et al. (2017). K25-A2, K26-A2, K29-A2, and K31-A2 strains are resistant to clavulanic acid-amoxicillin (AMC), amoxicillin (AMO), ATM, CAZ, cefoxitin (CFO), CPM, cefotaxime (CTX), CIP, LVX, nalidixic acid (NAL), norfloxacin (NOR), piperacillin/tazobactam (PIT), and trimethoprim/sulfamethoxazole (SUT). K29-A2 and K31-A2 strains are also resistant to ertapenem (ERT), IMP, and MEM. Of the four clinical bacterial isolates evaluated in this study, three strains of *K. pneumoniae* showed moderate biofilm production (K-26-A2, K-29-A2, and K-31-A2,) and one strong (K-25-A2) (Scavuzzi et al. 2017).

After isolation, identification, and characterization in the study by Scavuzzi et al. (2017), the strains were transported to the Clinical Microbiology Sector of the Keizo Asami Immunopathology Laboratory of the Federal University of Pernambuco (iLIKA/UFPE), preserved in glycerol at − 80 °C.

### Evaluation of biofilm formation inhibition

To evaluate the inhibition of biofilm formation, tryptone soy broth (TSB) + glucose was initially distributed in each well of the microdilution plates. Then, the NEs and CUR were added at the concentrations of MIC, MIC/2, MIC/4, MIC/8, and MIC/16, and, later, bacterial suspensions (10^5^ CFU/mL) of *K. pneumoniae* K25-A2 were added, K26-A2, K29-A2, and K31-A2. The microplates were incubated at 35 ± 2 °C for 24 h. TSB without inoculum was used as a sterility control (negative control); TSB with inoculum was used as a microbial growth control (positive control). After incubation, the inhibition of biofilm production was quantified by the crystal violet method (Stepanovic et al. 2000), and the results were expressed as minimum biofilm inhibitory concentration (MBIC) considering the percentage of inhibition greater than or equal to 80% (Lima et al. 2017).

### Assessment of biofilm eradication

Assays to assess biofilm eradication were performed with clinical isolates from the previous study. Initially, bacteria were adjusted to a density of 0.5 on the McFarland scale in TSB + glucose and distributed in microdilution plates. Plates were incubated at 35 ± 2 °C for 24 h to allow biofilm formation. After incubation, the culture medium was removed and replaced with a new medium. Then, NEs and CUR were distributed in microplates at concentrations of 16 × MIC, 8 × MIC, 4 × MIC, 2 × MIC, and MIC and incubated at 35 ± 2 °C for 24 h. TSB without inoculum was used as a sterility control (negative control); TSB with inoculum was used as a microbial growth control (positive control). After incubation, biofilm eradication was quantified by the crystal violet method (Stepanovic et al. 2000), and the results were expressed as minimum biofilm eradication concentration (MBEC) and percentage of inhibition (Lima et al. 2017).

### Statistical analysis

Statistical analysis was used to identify the antibacterial potential of NEs containing CUR, through regression, correlation analysis, graphs, and tables elaboration, aiming to understand the behavior of the variables. The data obtained were statistically analyzed and expressed as mean ± standard deviation (SD). The significance level used to reject the null hypothesis was 0.05.

## Results

### Experimental design of olive oil nanoemulsions containing curcumin from *Curcuma longa* L.

Intending to develop a formulation with attractive characteristics for both the food and pharmaceutical industries, the proposed oil for the oily phase was the olive oil (*Olea europaea* L.). In addition to the oil, it was also observed the HLB to be obtained with the use of different surfactants (Tween 80—15; Tween 60—14.9; Tween 40—15.6; Tween 20—16.7; Span 80—4.3; Span 20—8.6) so that an ideal HLB for the formulation could be obtained. An HLB between 8 and 18 is valid for O/W emulsifiers, and an HLB between 10 and 18 is valid for solubilizers.

In this sense, various combinations of different percentages of the surfactants were calculated for mixing with the olive oil. From 0.5 to 7% of each surfactant were calculated, being observed that the HLB of the proportion obtained with 5% of tween 80 and 2.5% of span 80 fits the previously listed characteristics, obtaining an HLB of 11.43, similar to the HLB of the oil. In this regard, this proportion was used to proceed with the theoretical studies of the best composition of NE. In addition to the theoretical evaluation, olive oil and selected surfactants were included in the following proportion: olive oil—2.5 to 5%, tween 80—2.5 to 5%, Span 80—2.5 to 5%, filling up with water, to obtain some formulations for a better definition of a system prototype and for choosing the best composition of NEs (Table 1).

**Table 1:** Macroscopic evaluation of the obtained nanoemulsions

Formulation	Macroscopic characteristics	Stability (days)
1 (Tween 80 = 2.5%; Olive oil = 2.5%, Span 80 = 2.5%)	White, milky, fluid formulation + + + +	7 d
2 (Tween 80 = 5%; Olive oil = 2.5%, Span 80 = 2.5%)	Opaque yellow, milky, slightly oily formulation with oil on the surface	2 d
3 (Tween 80 = 2.5%; Olive oil = 5%, Span 80 = 2.5%)	Opaque yellow, milky, fluid formulation + +	7 d
4 (Tween 80 = 5%; Olive oil = 5%, Span 80 = 2.5%)	White, milky, fluid formulation + + + +	> 120 d
5 (Tween 80 = 2.5%; Olive oil = 2.5%, Span 80 = 5%)	White opaque, milky fluid formulation + +	5 d
6 (Tween 80 = 5%; Olive oil = 2.5%, Span 80 = 5%)	Opaque white, milky, slightly oily formulation with oil on the surface	10 d
7 (Tween 80 = 2.5%; Olive oil = 5%, Span 80 = 5%)	Opaque yellowish, fluid milky formulation +	5 d
8 (Tween 80 = 5%; Olive oil = 5%, Span 80 = 5%)	Yellowish opaque, milky, slightly oily formulation with oil on the surface	2 d

Mixing another surfactant with Tween 80 can cause the hydrophilic and lipophilic properties to balance, which is why to compose the surfactant mixture, Span 80 was added. The calculation of the final HLB value, according to the proportion of surfactant to be added, was carried out according to Eq. 2.

2 $${\text{HLB}}({\text{X}})=({\text{HLB}}({\text{A}}) \times 0.01 \times \mathrm{ A }+\mathrm{ HLB}({\text{B}}) \times 0.01 \times \mathrm{ B})$$

where HLB(X) is the HLB value resulting from surfactant mixtures, A is the percentage of hydrophilic surfactant, and B is the percentage of lipophilic surfactant, where A + B = 100, HLB(A), and HLB(B) correspond to the HLB of Tween 80 and Span 80, respectively. In this sense a factorial analysis for choosing the best nanoemulsion composition was performed. The nanoformulations obtained were evaluated macroscopically, as shown in Table 1.

At the end of the macroscopic evaluation, olive oil and selected surfactants were included in the following proportion for an ideal NE: olive oil—5%, Tween 80—5%, and Span 80—2.55%. Therefore, this proportion was followed for the design of the best production process. NEs were produced in two different ways: at room temperature (NE-1, NE-3, and NE-5) and heated to approximately 40 °C throughout the process (NE-2, NE-4, and NE-6).

**Fig. 1:**
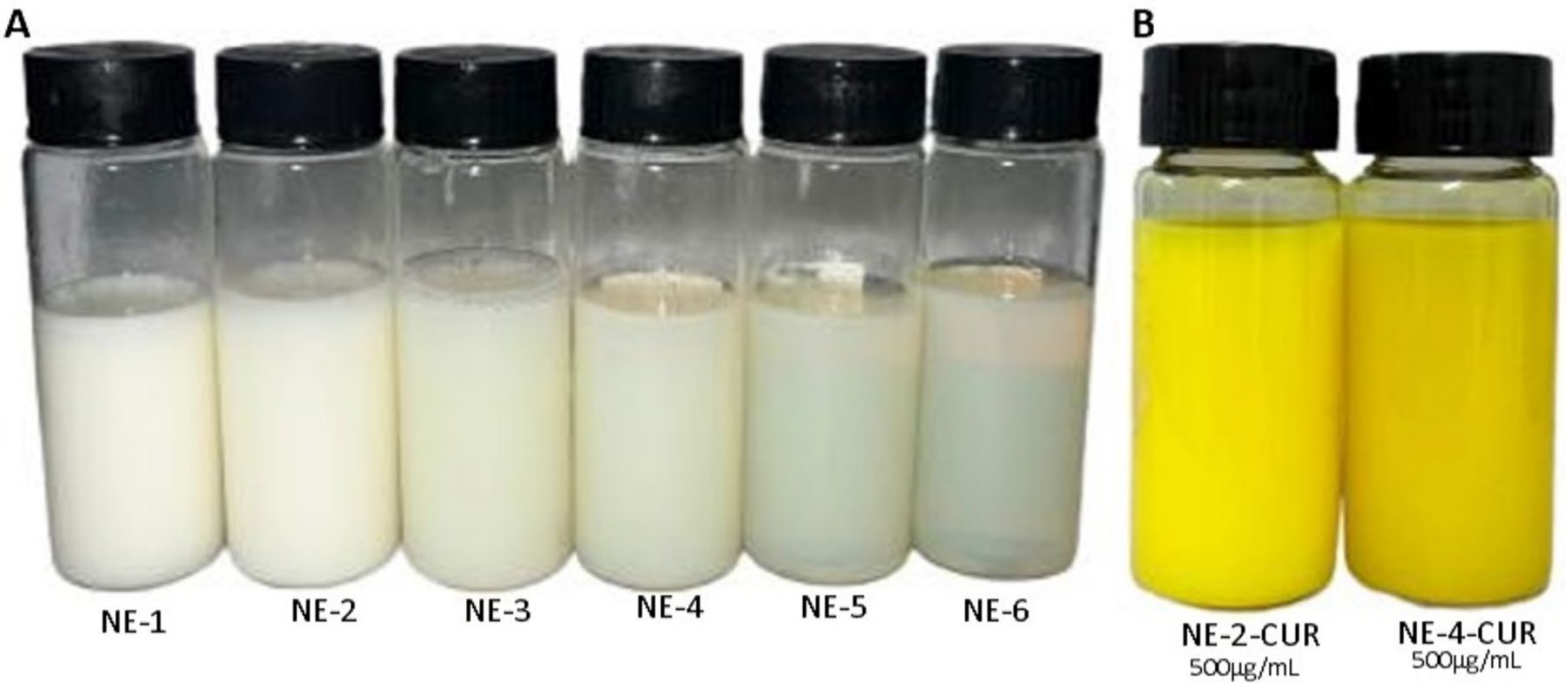
Nanoemulsions prepared in the experimental design

Another characteristic evaluated was the sonication time, that is, how long the formulation remained in the ultrasound probe. It was observed that the NEs obtained presented a whitish color, and the longer the ultrasound time, the clearer and more characteristic (Fig. 1). This characteristic corroborated for the *Ø*, the longer the probe, the smaller the particle sizes obtained (Table 2). Figure 1 shows some NEs tested in this study.

**Table 2:** Characterization of nanoemulsions

Formula	Particle size *Ø* (nm)	*PDI*	Zeta potential *ζ* (mV)	Macroscopic characteristics
NE-1	213.40 ± 5.63^a^	0.280 ± 0.09^ h^	− 32.40 ± 1.12^ m^	Milky, fluid, opaque white
NE-2	162.65 ± 13.91^b^	0.260 ± 0.02^ h^	− 29.00 ± 1.34^ m,n^	Clear, fluid, white, bluish effect
NE-3	156.90 ± 54.75^c^	0.381 ± 0.08^i^	− 28.95 ± 3.80^n^	Milky, fluid, opaque white
NE-4	77.15 ± 2.47^d^	0.417 ± 0.04^i^	− 25.70 ± 2.74^n,o^	Clear, fluid, white, bluish effect
NE-5	55.15 ± 1.91^e^	0.407 ± 0.01^i^	− 23.55 ± 1.32^o^	Clear, fluid, bluish effect, translucent
NE-6	42.30 ± 5.87^f^	0.341 ± 0.04^j^	− 23.80 ± 4.10^n,o^	Clear, fluid, bluish effect, translucent
NE-2-CUR (500 µg/mL)	165.40 ± 2.56^b^	0.254 ± 0.00^ k^	− 33.20 ± 1.35^p^	Yellowish, fluid, bluish effect, translucent
NE-4-CUR (500 µg/mL)	105.70 ± 4.13^ g^	0.459 ± 0.02^ l^	-32.10 ± 1.45^p^	Yellowish, fluid, bluish effect, translucent

Values are means ± SD of at least two experiments. Each experiment was done in triplicate. Different letters indicate that the results were not similar after a one-way analysis of variance followed by Bonferroni’s test for *p* < 0.05. NE-1: Nanoemulsion 1; NE-2: Nanoemulsion 2; NE-3: Nanoemulsion 3; NE-4: Nanoemulsion 4; NE-5: Nanoemulsion 5; NE-6: Nanoemulsion 6; NE-2-CUR: Nanoemulsion 2 containing curcumin from *C. longa*; NE-4-CUR: Nanoemulsion 4 containing curcumin from *C. longa*

The NE-1, NE-3, and NE-5 formulas were produced at room temperature, and the NE-2, NE-4, and NE-6 formulas were produced with heating throughout the experiment. The formula number refers to the probe time that the formulas were submitted (1—10 min, 2—20 min, and 3—30 mi). NE-1: Nanoemulsion 1; NE-2: Nanoemulsion 2; NE-3: Nanoemulsion 3; NE-4: Nanoemulsion 4; NE-5: Nanoemulsion 5; NE-6: Nanoemulsion 6.

The NEs developed in this study presented *Ø* between 42.30 ± 5.87 and 213.40 ± 5.63 nm (Table 2).

It was also observed that the NEs presented negative *ζ* and lower *PDI* values the shorter the ultrasound time. Regarding the size distribution of the systems, it is observed that it has three patterns with sizes greater than 100 nm and three patterns with sizes smaller than 100 nm. Considering, therefore, the analysis of the characteristics of the nanoemulsions obtained, NE-2 and NE-4 (both prepared by heating) were selected for the remaining experiments: NE-2 with 10-min probe and NE-4 20 min, therefore allowing the comparison of the results between more than one formula.

### Olive oil nanoemulsions containing curcumin from *Curcuma longa* L. (CUR)

From the encapsulation of CUR in the formulas NE-2 and NE-4, two NEs were obtained, NE-2-CUR and NE-4-CUR, respectively. Initially, the encapsulation of 1000 to 3000 µg/mL was attempted, not being successful, as there was precipitation. Then, a NE with 500 µg/mL of CUR was prepared (Fig. 1), obtaining a stable system without precipitation of the active ingredient. Also, the pH obtained for the NEs (NE-2-CUR: pH = 6.49; NE-4-CUR: 6.40) is similar to blood plasma, when comparing to the values referred by Kellum (2000). 

When evaluating the NEs in the zetasizer, it was observed that the NE-2-CUR has a slightly larger *Ø* (165.40 ± 2.56 nm) and that the *PDI* was 0.254, while NE-4-CUR presented a smaller size (105.70 ± 4.13 nm), but a larger *PDI* (0.459) (Table 2).

### Scanning electron microscopy

**Fig. 2:**
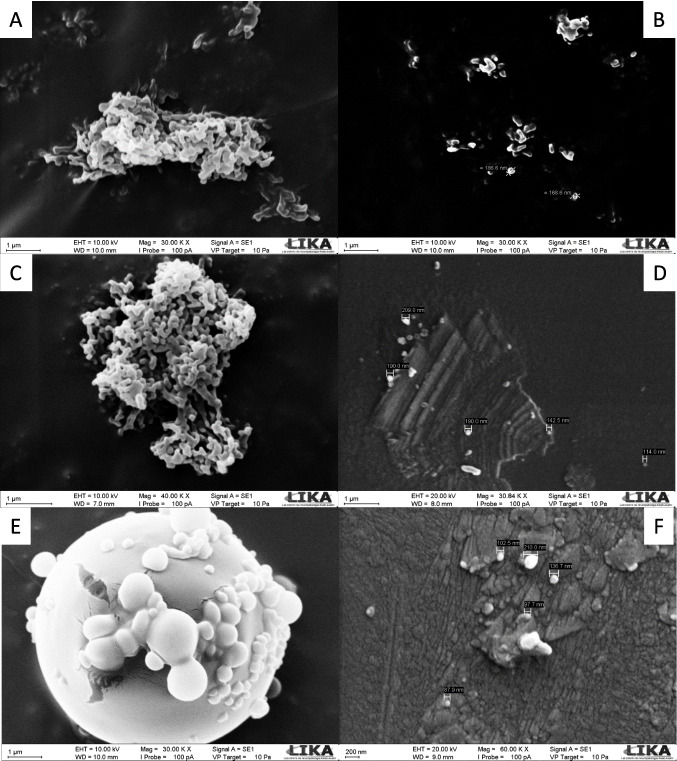
Morphology of nanoemulsions obtained by scanning electron microscopy

The images obtained by SEM show a spherical morphology, a smooth surface, and a homogeneous population of NEs; NE-2 and NE-2-CUR have a similar shape, as well as NE-4-CUR, but NE-4-CUR has a slightly more heterogeneous size (Fig. 2).

NE-2 (A and B), NE-2-CUR (C and D), and NE-4-CUR (E and F) obtained by scanning electron microscopy. Images A, B, D, E at 30.00 KX magnification. Image C at 40.00 KX magnification. Image F at 60.00 KX magnification. NE-2: Nanoemulsion 2, without curcumin; NE-2-CUR: Nanoemulsion 2 containing curcumin from *C. longa*; NE-4-CUR: Nanoemulsion 4 containing curcumin from *C. longa*.

### Stability and entrapment drug efficiency of nanoemulsions

**Fig. 3:**
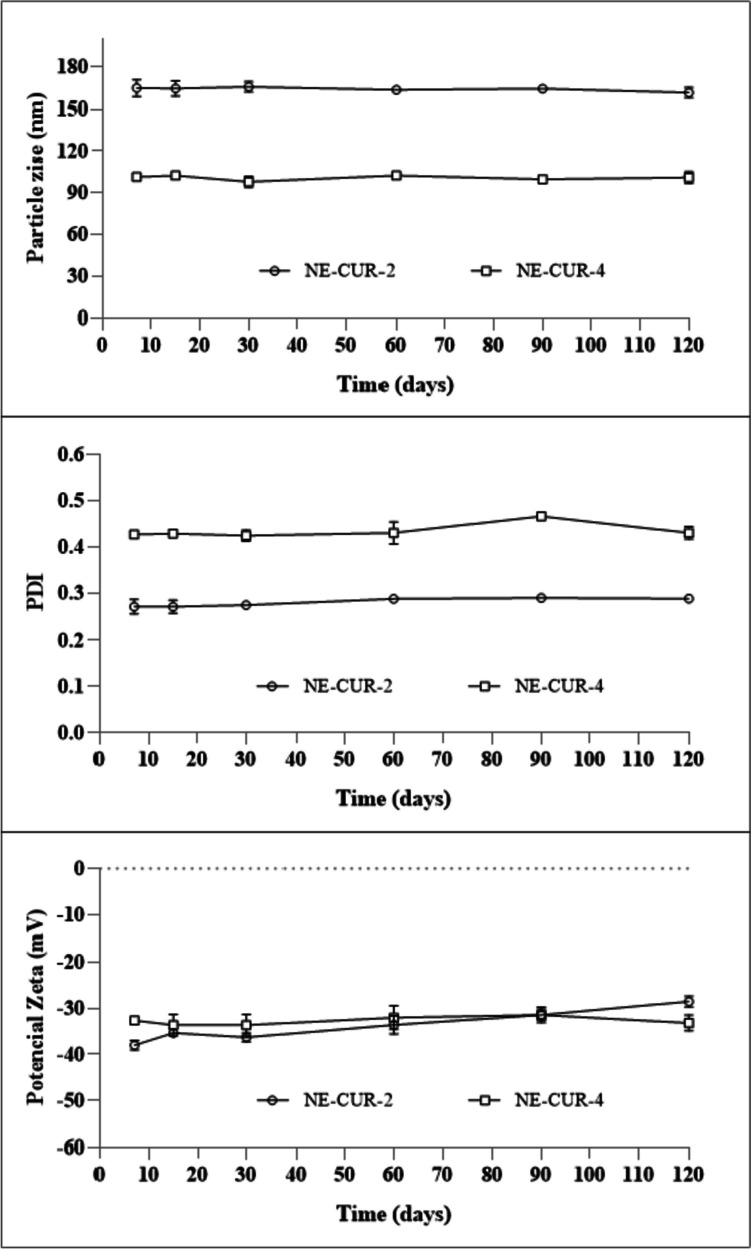
Graphics of nanoemulsion stability NE-2-CUR and NE-4-CUR

In the stability studies, it was found that the parameters (*PDI*, *Ø*, and *ζ*) remained stable during the 120-day period in which the NEs were evaluated (Fig. 3). Furthermore, both NEs were efficient systems to encapsulate CUR (NE-2-CUR: 99% and NE-4-CUR: 99.28%).

NE-2-CUR: Nanoemulsion 2 containing curcumin from *C. longa*; NE-4-CUR: Nanoemulsion 4 containing curcumin from *C. longa*.

### Differential scanning calorimetry (DSC) and thermogravimetry (TG)

**Fig. 4:**
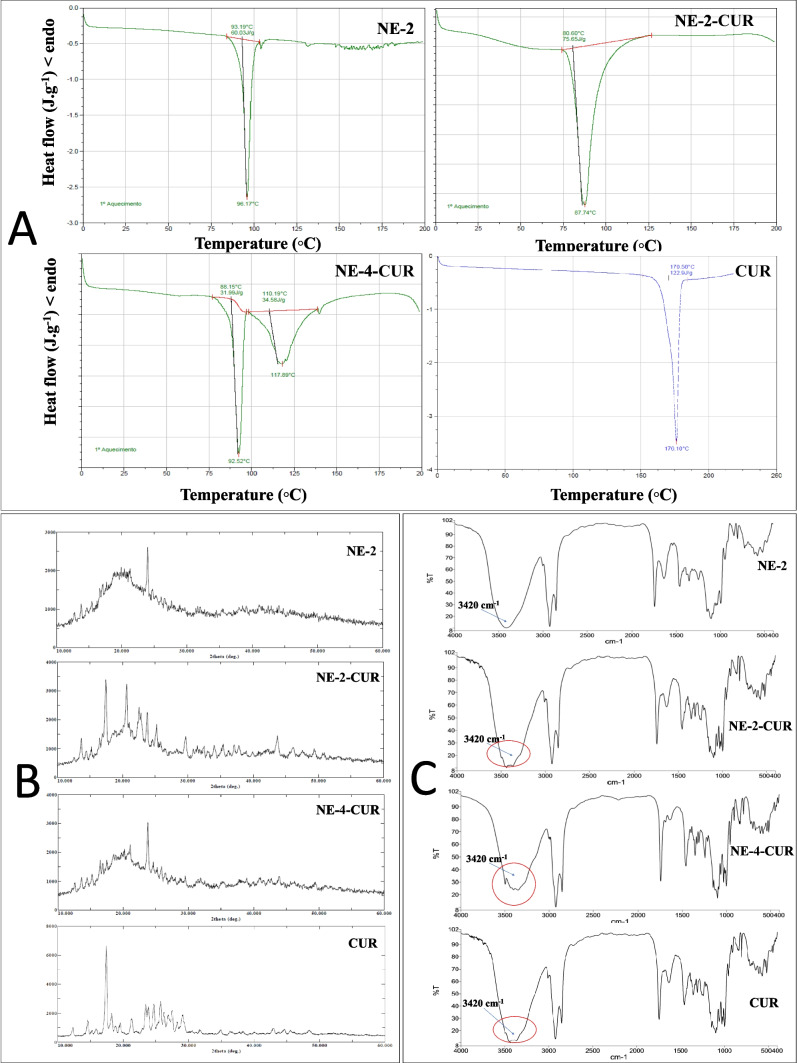
Physicochemical characterization of nanoemulsions and their components

The TG curves for the evaluated NEs showed that the thermal decomposition occurred in three stages (Table 3, Fig. 4 and figures S1 to S4). In the first stage, the mass loss of NE-2 was 3.09%, while NE-2-CUR and NE-4-CUR were 6.01 and 6.11%, respectively. There was no significant change in the percentage of mass lost by each sample. The mass loss of the CUR also presented three stages of decomposition; however, the percentage of loss in the first stage (37–198 °C) was only 0.68%, showing a loss of 5 to 6% of other substances that make up the NEs.

**Table 3:** TG and DSC data for nanoemulsions

TG			
Sample	Phases	Start-ending (°C)	Mass loss (%)
NE-2	1	69.32–150.32	3.09
	2	175.00–414.68	78.90
	3	414.68–521.32	14.46
NE-2-CUR	1	91.17–173.23	6.07
	2	173.23–409.34	76.44
	3	409.34–547.20	13.90
NE-4-CUR	1	94.18–175.00	6.01
	2	175.00–420.84	77.09
	3	420.84–548.83	13.69
CUR	1	37.16–198.00	0.68
	2	200.04–363.01	37.09
	3	367.01–575.00	62.28
DSC			
Sample	Event	Peak (°C)	ΔH (J/g^−1^)
NE-2	1	96.17	60.03
NE-2-CUR	1	87.74	75.65
NE-4-CUR	1	92.52	31.99
	2	117.89	34.58
CUR	1	176.10	122.9

CUR: curcumin from *C. longa* (pure curcumin); NE-2: Nanoemulsion 2, without curcumin; NE-2-CUR: Nanoemulsion 2 containing curcumin from *C. longa*; NE-4-CUR: Nanoemulsion 4 containing curcumin from *C. longa*

This also shows that part of the other components of NEs are decomposed in this second stage, along with curcumin. In the third stage, the pure CUR suffers a loss of 62.28%, while the NEs suffer greater losses between 13.69 (NE-4-CUR) and 14.46% (NE -2) (Table 3).

Regarding the second stage of decomposition, the CUR showed a loss of approximately 37.09% and the NEs of 76.44 (NE-2-CUR) and 78.90% (NE-2). This also shows that part of the other components of NEs are decomposed in this second stage, along with curcumin. This also shows that part of the other components of NEs are decomposed in this second stage, along with curcumin. In the third stage, the pure CUR suffers a loss of 62.28%, while the NEs suffer greater losses between 13.69 (NE-4-CUR) and 14.46% (NE-2) (Table 3). Therefore, it is evident that NE-2-CUR and NE-4-CUR followed a NE-2 decomposition profile, which represents only the NE components without CUR since the values presented in the three steps are very similar with the one presented by NE-2. This fact could be related to a possible change of CUR from a crystalline state to an amorphous state in samples NE-2-CUR and NE-4-CUR.

Table 3 shows the DSC results. The endothermic peak is related to the melting point, which occurred around 176.10 °C. ^41^ It can be observed in the NE samples the absence of the CUR peak; this means that the curcumin was completely inside the nucleus of NEs and in greater concentration in the amorphous form, which would explain its non-detection. The NEs showed melting events of 87.74 °C (NE-2-CUR), 92.52 °C (NE-4-CUR), and 96.17 °C (NE-2) which are related to components of the NEs such as Twenn® (Fig. 4).

**A**: Differential scanning calorimetry (DSC); **B**: thermogravimetry (TG); **C**: Fourier transform infrared spectroscopy (FTIR). CUR: curcumin from *C. longa* (pure curcumin); NE-2: Nanoemulsion 2, without curcumin; NE-2-CUR: Nanoemulsion 2 containing curcumin from *C. longa*; NE-4-CUR: Nanoemulsion 4 containing curcumin from *C. longa*.

### X-ray diffraction analysis

XRD analyses were carried out to evaluate possible changes in the crystallinity of the components present in the produced NEs. Figure 4 shows the diffractograms of samples NE-2, NE-2-CUR, and NE-4-CUR.

The characteristic peaks of CUR, according to Meng et al. (2020) at 8.83, 12.23, 14.48, 17.17, 23.21, 24.64, 25.61, and 28.90° are indicative of its highly crystalline nature. NE-2-CUR showed the characteristic peaks of pure CUR at 13.70, 17.45, 22.55, 23.85, 25.25° and with the absence of other peaks such as 8.83 and 28.90°, which shows a decrease in the crystallinity of the CUR in the sample.

Regarding the peaks of the NE-4-CUR sample, the characteristic peaks of the CUR were not observed, with the diffractogram being practically identical to that of the NE-2, which only represents the other components without the CUR.

### Fourier transform infrared spectroscopy (FTIR)

FTIR was used to confirm the presence of specific functional groups present in NEs containing CUR and a possible interaction of its components (Fig. 4).

The main CUR peaks, such as C–O elongation at 1206 cm^−1^, trans CH–CH elongation, at 1026 cm^−1^, and asymmetric C–O–C elongation, at 856 cm^−1^,^42^ were visible in the NE-2-CUR and NE-4-CUR spectra (Fig. 4). It can be also observed in NE-2-CUR and NE-4-CUR presented peaks attributed to the presence of these functional groups without any alteration.

### Evaluation of antibacterial activity

The MIC of the NEs developed against all evaluated ATCC bacteria varied between 125 and 250 µg/mL, while the MIC of the CUR was > 250 µg/mL for all evaluated bacteria (Table 4). The most sensitive bacteria were *S. aureus* ATCC 25923, *S. aureus* ATCC 33591, and *E. faecalis* ATCC 29212, for which NE-2-CUR showed a MIC of 125 µg/mL. For the other evaluated bacteria, *E. coli* ATCC 13846, *E. coli* 25,922, *K. pneumoniae* ATCC 13883, *K. pneumoniae* ATCC 700603, *A. baumannii* ATCC 19606, and *P. aeruginosa* ATCC 27853, NE-2-CUR and NE-4 -CUR showed MIC of 250 µg/mL.

**Table 4:** Evaluation of the antibacterial activity of curcumin from *Curcuma longa* L. (CUR) and NE-2-CUR and NE-4-CUR nanoemulsions

	*S. aureus* ATCC 25923	*S. aureus* ATCC 33591	*E. faecalis* ATCC 29212	*E. coli* ATCC 13846	*E. coli* ATCC 25922	*P. aeruginosa* ATCC 27,853	*A. baumannii* ATCC 19,606	*K. pneumoniae* ATCC 13,883	*K. pneumoniae* ATCC 700,603	K-25-A2	K-26-A2	K-29-A2	K-31-A2
MIC (µg/mL)													
NE-2-CUR	125	125	125	250	250	250	250	250	250	250	250	250	250
NE-4-CUR	250	250	250	250	250	250	250	250	250	250	250	250	250
CUR	250	250	> 250	250	> 250	> 250	> 250	> 250	> 250	512	> 512	512	512

*ATCC* American Type Culture Collection, *MIC* minimum inhibitory concentration, *CUR* curcumin from *Curcuma longa* L., *NE-2-CUR* nanoemulsion 2 containing curcumin, *NE-4-CUR* nanoemulsion 4 containing curcumin, *K-25-A2, K-26-A2, K29-A2, and K-31-A2* clinical isolates of *Klebsiella pneumoniae* resistant to antibiotics and biofilm and efflux pump producers

The NEs evaluated in the present study are promising against these Gram-positive bacteria, considering that a MIC of 125 µg/mL was observed against *S. aureus* and *E. faecalis*. In this way, it was possible to observe that the NEs potentiated the antibacterial activity of CUR against the evaluated bacteria.

Thus, the NEs were also evaluated against the bacteria K25-A2, K26-A2, K29-A2, and K31-A2, which are clinical isolates of *K. pneumoniae* and are efflux pump and biofilm producers from hospitals in Recife-PE, from the study by Scavuzzi et al. (2017).

In the present study, for these resistant clinical isolates, MICs of 250 µg/mL were verified, so that the encapsulation of CUR in NEs proved to be a promising strategy to potentiate the effects of this compound. It should be noted that these isolates are resistant to clavulanic acid-amoxicillin (AMC), amoxicillin (AMO), ATM, CAZ, cefoxitin (CFO), cefotaxime (CTX), CIP, LVX, nalidixic acid (NAL), norfloxacin (NOR), piperacillin/tazobactam (PIT), and trimethoprim/sulfamethoxazole (SUT). K29-A2 and K31-A2 strains are also resistant to ertapenem (ERT), IMP, and MEM. Therefore, drugs and other substances with the potential to combat them are of great relevance.

Therefore, the encapsulation of CUR in NEs is a promising therapeutic alternative, as it potentiates the antibacterial activity and enables administration by oral, nasal, transdermal, or intravenous routes for the treatment of intestinal, respiratory, skin, and systemic infections caused by *S. aureus*, *E. faecalis*, *A. baumannii*, *E. coli*, *K. pneumoniae*, and *P. aeruginosas*, as well as antibiotic-resistant strains of *K. pneumoniae* (Table 4).

### Evaluation of the antibacterial activity of nanoemulsions in association with ceftazidime against clinical isolates of resistant *Klebsiella pneumoniae*

The NEs containing CUR were found to predominantly have a synergistic or additive profile with CAZ (Table 5). The interaction of CAZ with NE-2-CUR showed synergism for *K. pneumoniae* ATCC 13883, K-26-A2, K-31-A2, and an additive profile for K-25-A2 and K29-A2. Only the interaction between NE-4-CUR and CAZ was indifferent to the K-29-A2 and K-31-A2 bacteria, although for the other bacteria (*K. pneumoniae* ATCC 13883, K-25-A2, and K- 26-A2) synergism was verified.

**Table 5:** Evaluation of the interaction between ceftazidime and nanoemulsions NE-2-CUR and NE-4-CUR against *Klebsiella pneumoniae*

Bacteria	CAZ	CUR	NE-2-CUR	NE-4-CUR	CAZ + CUR/NE			FICI			Interaction		
MIC (µg/mL)					MIC of the combination (µg/mL)								
					CAZ + CUR	CAZ + NE-2-CUR	CAZ + NE-4-CUR	CAZ + CUR	CAZ + NE-2-CUR	CAZ + NE-4-CUR	CAZ + CUR	CAZ + NE-2-CUR	CAZ + NE-4-CUR
*K. pneumoniae* ATCC 13883	32	> 512	250	250	0.25/32	4/7.81	4/7.81	0.07	0.15	0.15	Synergism	Synergism	Synergism
K-25-A2	256	512	250	250	2/32	4/125	32/62.5	0.07	0.51	0.37	Synergism	Additive	Synergism
K-26-A2	64	> 512	250	250	2/16	16/15.63	0.25/7.81	0.03	0.31	0.04	Synergism	Synergism	Synergism
K-29-A2	16	512	250	250	8/8	0.5/125	0.25/250	0.51	0.53	1.01	Additive	Additive	Indifferent
K-31-A2	32	512	250	250	32/8	0.25/62.5	0.25/250	1.01	0.28	1.00	Indifferent	Synergism	Indifferent

*ATCC* American Type Culture Collection, *CAZ* ceftazidime, *MIC* minimum inhibitory concentration, *CUR* curcumin from *Curcuma longa* L., *NE-2-CUR* nanoemulsion 2 containing curcumin, *NE-4-CUR* nanoemulsion 4 containing curcumin, *K-25-A2, K-26-A2, K29-A2, and K-31-A2* clinical isolates of *Klebsiella pneumoniae* resistant to antibiotics and biofilm and efflux pump producers

It was found that, for those strains in which the association showed a synergistic or additive profile, there was a reduction in CAZ MICs between 256 times (K26-A2) and 2 times (K29-A2). For example, for K25-A2, it was possible to verify a 64-fold reduction in the CAZ MIC from 256 to 4 µg/mL when associated with NE-2-CUR and an eightfold reduction in the CAZ MIC from 256 to 32 µg /mL when associated with NE-4-CUR. Therefore, it is observed that the association of NEs produced with ceftazidime can be a promising strategy in the fight against diseases caused by these bacteria.

### Evaluation of antibiofilm activity against resistant clinical isolates of *Klebsiella pneumoniae*

Combating biofilm-producing bacteria is a problem, and products that have the potential to inhibit their formation are needed. The NEs produced in the present study inhibited the formation of biofilms in the evaluated clinical isolates. The NEs showed MBIC ranging from 15.625 to 250 µg/mL (Table 6); however, no activity of curcumin alone was observed. In this sense, it is observed that the encapsulation of this bioactive compound reverses this situation, probably due to the properties of CUR, which hinder the action of this compound (hydrophobicity, rapid degradation, photosensitivity, among others).

**Table 6:** Antibiofilm activity of curcumin from *Curcuma longa* L. (CUR) and nanoemulsions NE-2-CUR and NE-4-CUR against clinical isolates of *Klebsiella pneumoniae*

Bacteria	CUR	NE-2-CUR	NE-4-CUR	CUR	NE-2-CUR	NE-4-CUR
	MBIC (µg/mL)			MBEC (µg/mL)		
K-25-A2	> 250	62.5	125	> 250	> 250	> 250
K-26-A2	> 250	31.25	31.25	> 250	> 250	> 250
K-29-A2	> 250	31.25	31.25	> 250	> 250	> 250
K-31-A2	> 250	15.625	15.625	> 250	> 250	> 250

*MBIC* minimum biofilm inhibitory concentration, *MBEC* minimum biofilm eradication concentration, *CUR* curcumin from *Curcuma longa* L., *NE-2-CUR* nanoemulsion 2 containing curcumin, *NE-4-CUR* nanoemulsion 4 containing curcumin, *K-25-A2, K-26-A2, K29-A2, and K-31-A2* clinical isolates of *Klebsiella pneumoniae* resistant to antibiotics and biofilm and efflux pump producers

Although the eradication of biofilms was not observed with the NEs tested in the present study (MBEC ˃ 250 µg/mL) (Table 6), the capacity of the nanotechnological products developed in this study to prevent the formation of the biofilm is a relevant characteristic, as biofilm-producing microorganisms contribute to the wide dissemination of multi-drug resistant strains, as these bacteria in the biofilm share resistance genes, contributing to the increase of these pathogens in the unit.

## Discussion

### Experimental design of olive oil nanoemulsions containing curcumin from *Curcuma longa* L.

One important factor for NEs is the HLB of different surfactants (Tween 80—15; Tween 60—14.9; Tween 40—15.6; Tween 20—16.7; Span 80—4.3; Span 20—8.6); in this sense, an ideal HLB for the formulation was desired. An HLB between 8 and 18 is valid for O/W emulsifiers, and an HLB between 10 and 18 is valid for solubilizers (Gadhave 2014).

Griffin developed an arbitrary scale of values that serve as a measure of the HLB of superficially active agents. Therefore, through this numerical system of the HLB index, an interval of maximum efficiency can be defined for each type or class of surfactant. On this scale, the higher the HLB of an agent, the more hydrophilic it will be (Griffin 1949). Thus, it is known that the use of Tween 80 (HLB = 15) is categorized as an O/W surfactant that has a higher solubility in the aqueous phase than in the organic phase (Sahlan et al. 2018). It is recognized that Tween 80 contains a lipophilic polysorbate group facing the lipophilic core of the curcumin micelle and a hydrophilic polyoxyethylene group facing hydrophilic solvents (Aly et al. 2017), making it suitable for use to encapsulate curcumin from *Curcuma longa* L. (CUR).

Also, the HLB of the proportion of 5% of tween 80 and 2.5% of span 80 is similar to the HLB of the oil. In this regard, this proportion was used to proceed with the theoretical studies of the best composition of NE. Mixing another surfactant with Tween 80 can cause the hydrophilic and lipophilic properties to balance, so that both surfactants will tend to be more at the interface and slightly soluble in the bulk phase (Sahlan et al. 2018), which is why to compose to the surfactant mixture, Span 80 was added. Furthermore, it is important for NE that the proportion of surfactants results in an HLB similar to that of oil, which for olive oil is 12 (Frange and Garcia 2009). Therefore, an HLB of 12 was considered the ideal result for the proportion of surfactants to be included in the NE.

At the end of the macroscopic evaluation, olive oil and selected surfactants were included in the following proportion for an ideal NE: olive oil—5%, Tween 80—5%, and Span 80—2.5. Therefore, NEs were produced different temperature conditions. Temperature affects the physical properties of the oil, water, interfacial films, and solubilities of surfactants in the oil and water phases, consequently affecting the stability of the emulsion. The most important effect of temperature seems to be on the viscosity of emulsions, given that viscosity decreases with increasing temperature, mainly due to a decrease in oil viscosity; in the same way, temperature increases the thermal energy of the system and, therefore, increases the frequency of particle collisions (Aljabri et al. 2022; Sahlan et al. 2018; Sadurní et al. 2005).

Also, temperature helps the solubility of CUR at the time of incorporation into systems, with an increase of up to 12 times in solubility when heating CUR compared to unheated CUR, not affecting the stability of curcumin (Kurien et al. 2007); due to these characteristics, the process that was performed in a heating manner was considered better.

Another characteristic evaluated was the sonication time, which corroborated for the *Ø*; the longer the probe, the smaller the particle sizes obtained. The use of ultrasound is a widely used high-energy method to reduce the size of NEs particles. In this method, mechanical vibrations of ultrasound waves (> 20 kHz) create sinusoidal pressure variation in the emulsion system (Gupta et al. 2015). This processing leads to microjet impacts and shock waves and collisions between particles, resulting in size reduction (Aljabri et al. 2022; Franco et al. 2004).

According to Franco (2004), the particle size first decreases exponentially with increasing ultrasound time; however, it tends to be stationary after certain minutes. Therefore, it is not necessary to keep increasing the processing time to obtain the smallest particle size. In this sense, the NEs that need less sonication time were considered better for industry and for smaller particle sizes, and then, selected.

Regarding the *Ø*, *PDI*, *ζ*, these are fundamental parameters to determine the stability and potential of the formulations for application in in vivo and clinical tests. The NEs developed in this study presented *Ø* between 42.30 ± 5.87 and 213.40 ± 5.63 nm. It was also observed that the NEs presented negative *ζ* and lower *PDI* values the shorter the ultrasound time. High *ζ*, positive or negative, such as those obtained by NE-1 and NE-2, have been shown to contribute greatly to the stability of microemulsions and NEs due to highly charged surfaces, which resist droplet aggregation (Azami et al. 2018).

### Olive oil nanoemulsions containing curcumin from *Curcuma longa* L. (CUR) and scanning electron microscopy

NE-2 and NE-4 (both prepared by heating) were used for the remaining experiments and the production of the olive oil nanoemulsions containing curcumin from *Curcuma longa* L. (NE-2-CUR and NE-4-CUR, respectively), as the heating process as well as the shorter time of sonication was preferred. The NE with 500 µg/mL of CUR was prepared as a stable system without precipitation of the active ingredient, in addition to presenting pH like blood plasma (pH = 6.40–6.49).

Also, the NE-2 and the NE-4 demonstrated proper size, being valuable for multiple uses. In this sense, when evaluating the NEs containing CUR in the zetasizer, it was observed that the NE-2-CUR has a slightly larger *Ø*, while NE-4-CUR presented a smaller size, however, a larger *PDI*. Studies indicate that nanocarriers with sizes ranging from 50 to 200 nm are appropriate for intravenous applications, between 100 and 700 nm for nasal application, between 10 and 210 nm for transdermal applications via the transfollicular route, and between 50 and 500 nm for oral application (Danaei et al. 2018; Larese Filon et al. 2015; Mistry et al. 2009). The images obtained by SEM show a spherical morphology, a smooth surface, and a homogeneous population of NEs; NE-2 and NE-2-CUR have a similar shape, as well as NE-4-CUR, but NE-4-CUR with a slightly more heterogeneous size. In this sense, these NEs have potential use for applications using all possible routes of administration.

### Stability and entrapment drug efficiency of nanoemulsions

The *ζ* is correlated with the stability of the nanoparticle suspension, and values above + 30 mV or below − 30 mV indicate greater stability, due to the greater repulsion between the particles, reducing the aggregation between them (Danaei et al. 2018; Zhang et al. 2014); therefore, the *ζ* values obtained for the NEs corroborate the stability of the formulas. The emulsified systems are considered stable when they have an absolute value greater than 25 mV, so that when the *ζ* module is greater than 25 mV the repulsive forces predominate in relation to the London attractive ones, thus having a dispersed system. As for the *ζ* module lower than 25 mV, the attractive forces predominate in relation to the repulsive ones and the particles approach, generating flocculation and, consequently, phase separation (Lieberman et al. 1989).

Furthermore, both NEs were efficient systems to encapsulate CUR. Other authors, when evaluating NEs containing CUR, found lower Entrapment Drug Efficiency: 93.1% (Ngwabebhoh et al. 2018) and 80.9% (Gotmare et al. 2018). Thus, it is understood that NE-2-CUR and NE-4-CUR are stable NEs with physicochemical properties, as well as drug encapsulation capacity, with potential use in medicine.

### Differential scanning calorimetry (DSC) and thermogravimetry (TG)

Differential scanning calorimetry and thermogravimetry (DSC and TG) of solid samples provide results regarding endothermic and exothermic characteristics, melting point, crystallinity, polymorphism, and other thermal decomposition events. The NEs production process in this study promoted heating and homogenization phases capable of leading to possible changes in these properties, being important to evaluate the product using these techniques (Esquivel et al. 2022).

The TG curves for the evaluated NEs showed that there was no significant change in the percentage of mass lost by each sample, as according to Marcolino et al. (2011), the mass loss of the CUR also presented three stages of decomposition. Therefore, it is evident that NE-2-CUR and NE-4-CUR followed a NE-2 decomposition profile, which represents only the NE components without CUR since the values presented in the three steps are very similar to the one presented by NE-2. This fact could be related to a possible change of CUR from a crystalline state to an amorphous state in samples NE-2-CUR and NE-4-CUR.

The melting point range of curcumin should be around 184–190 °C (Ahmad et al. 2020). It can be observed in the NE samples the absence of the CUR peak; this means that the curcumin was completely inside the nucleus of NEs and in greater concentration in the amorphous form, which would explain its non-detection.

### X-ray diffraction analysis and Fourier transform infrared spectroscopy (FTIR)

XRD analyses were carried out to evaluate possible changes in the crystallinity of the components present in the produced NEs. NE-2-CUR showed peaks which shows a decrease in the crystallinity of the CUR in the sample. Regarding the peaks of the NE-4-CUR sample, the characteristic peaks of the CUR were not observed, with the diffractogram being practically identical to that of the NE-2, which only represents the other components without the CUR. It can be concluded that the preparation conditions of the NE-4-CUR sample were able to disperse the CUR more homogeneously in the nanodroplets, preventing its crystallization and allowing the CUR to remain in its amorphous and strongly encapsulated form. This fact makes the NE-4-CUR more interesting to release CUR in the medium, since actives in their amorphous form are more soluble than in their crystalline form (Budiman et al. 2023).

FTIR was used to confirm the presence of specific functional groups present in NEs containing CUR and a possible interaction of its components. The nanoemulsions containing curcumin presented the same peaks attributed to pure curcumin without any change, showing evidence of the absence of interactions between these specific groups.

### Evaluation of antibacterial activity

It is observed that CUR has strong antioxidant properties due to the active hydroxyl groups attached to the benzene rings (Tyagi et al. 2015), so these active hydroxyl groups respond quickly when they contact with reactive species, such as bacterial cells or cancer cells (Kumar et al. 2022). However, it is noteworthy that CUR, as a polyphenolic compound of lipophilic nature, when it encounters bacterial cells in an aqueous medium, has its bactericidal action strongly affected due to its high hydrophobicity. Therefore, the nanoformulation of hydrophobic components increases its potential to destroy bacteria due to stronger interaction with bacterial cells (Jiang et al. 2020).

It should be noted that bacterial infections cause high rates of morbidity and mortality, negatively impacting the world economy and public health. The therapy of these infections is expensive and prolonged, especially for the treatment of infections caused by gram-negative bacilli, such as *K. pneumoniae*, *E. coli*, and *P. aeruginosa* (Abdulrahman et al. 2020; Bassetti et al. 2019; Kofteridis et al. 2020), which were used in the present study.

It is worth noting that the bacteria used in this study are involved in infections of the urinary tract, digestive tract, central nervous system, intra-abdominal, skin, and wounds, in addition to causing pneumonia associated with mechanical ventilation, bacteremia, sepsis, and infections in the intensive care units (ICU) (Li et al. 2021; MacVane 2017).

In this regard, in relation to *P. aeruginosa*, this bacterium has natural and acquired resistance mechanisms that make the clinical management of the infected patient difficult. This is due to its intrinsic characteristics of presenting a low level of sensitivity to antimicrobials, due to the low permeability of its membrane, the ability to form biofilm and to carry plasmids and resistance genes (Abdulrahman et al. 2019; Nwabuife et al. 2022).

There are several mechanisms of acquired resistance, such as overexpression of efflux pumps, production of P-lactamases, and loss or reduced expression of outer membrane proteins (Abdulrahman et al. 2020; Hedayati et al. 2021). Therefore, it constitutes one of the paradigms of bacterial resistance, as it is a bacterium to which all resistance mechanisms can easily converge, being essential new therapeutic products.

Despite having commensal strains in the gastrointestinal tract, *E. coli* is also an important cause of infections. This bacterium presents a variety of intestinal and extraintestinal pathogenic strains capable of causing different infections with specific clinical conditions (Nwabuife et al. 2022). On the other hand, *A. baumannii* is a gram-negative bacterium that causes up to 20% of infections in intensive care units (ICUs) worldwide and is also adept at acquiring antimicrobial resistance, being classified as ‘high priority’ by WHO - World Health Organization (2017) and WHO - World Health Organization (2019). In ICUs, infections by this microorganism are usually associated with the process of mechanical ventilation and can reach the lungs and bloodstream (Sarshar et al. 2021).

As for Gram-positive bacteria, it is known that *S. aureus* colonizes the skin and nasal mucous membranes, and when it breaks the barriers of the immune system, it ceases to be a commensal bacterium and becomes a pathogen, capable of modifying itself and adapting to the host’s environment, establishing an infection (Guo et al. 2020). Strains of MRSA can produce a penicillin-binding protein (PBP), leading to a decreased affinity for most penicillins (Nandhini et al. 2022).

Therefore, although this bacterium is usually found on the skin or nose of about one-third of the world's inhabitants and normally causes minor skin infections in healthy individuals, when *S. aureus* becomes resistant to any antibiotic, it can cause serious infections or opportunistic diseases and be resistant to many antibiotics such as methicillin, penicillin, oxacillin, cloxacillin, cefazolin, cefoxitin, and common antibiotics (Mickymaray et al. 2018), being important the development of new strategies to overcome this problem.

Furthermore, it is known that the intestinal microbiota performs several functions in the human host and can directly influence health, affecting metabolism, the immune system, and hormone production (Chattopadhyay et al. 2021). Among the microorganisms that make up the intestinal microbiota, *E. faecalis* stands out. This Gram-positive bacterium is commensal to the gastrointestinal tract; however, it can act as an opportunistic pathogen, through the expression of virulence factors (Adak and Khan 2019.

Due to its importance and its relationship with several diseases, such as endocarditis, urinary tract infection, prostatitis, intra-abdominal infection, cellulitis, wound infection, irritable bowel syndrome, obesity, insulin resistance, ulcerative colitis, Crohn’s disease, depression, and neoplasms, the composition and function of the intestinal microbiota have been receiving attention and being the target of new discoveries in recent decades (Chattopadhyay et al. 2021; Madsen et al. 2017). Thus, it appears that the NEs evaluated in the present study are promising against these Gram-positive bacteria, considering that a MIC of 125 µg/mL was observed against *S. aureus* and *E. faecalis*.

In this way, it was possible to observe that the NEs potentiated the antibacterial activity of CUR against the evaluated bacteria. Furthermore, other nanoformulations containing curcumin were produced and evaluated against the same bacterial species used in the present study. The study by Reddy and collaborators (2020) also evaluated the potential of CUR encapsulated in nanoformulations against bacteria. In this study, cur-C3 and CD (α, β) inclusion complexes were prepared with different molar ratios (75% CUR, 16% demethoxycurcumin, and 4% bisdemethoxycurcumin) in β-cyclodextrin, with the complexes presenting MICs of 250 and 310 µg/mL against *E. coli* and *S. aureus*, respectively. That said, it is evident that the use of NEs is a valid strategy to encapsulate the CUR and improve the application of this substance against these pathogens.

Another nanoformulation evaluated against pathogenic bacteria was the thermoresponsive hydrogels produced by Alves and collaborators (2017); these hydrogels were synthesized with poloxamer 407 (P407), CUR solid dispersion (CUR-SD), and AgNPs obtained with sodium citrate (AgNP-citrate). When evaluated against strains of *S. aureus*, *P. aeruginosa*, and *E. coli*, an MIC of 400 µg/mL was obtained, concentrations higher than those found in the present study, corroborating that NEs are formulations that improve the activity of bioactive compounds such as the curcumin.

Furthermore, it is observed that *K. pneumoniae* is a bacterium that is commonly associated with resistant strains, mainly in hospitalized or immunocompromised patients (Confessor 2019; Scavuzzi et al. 2017). The treatment of infections caused by resistant strains has become a concern and the subject of studies, as they are common in hospitals and are associated with high morbidity and mortality rates, especially among patients with prolonged hospitalization exposed to invasive devices, requiring a strategic therapeutic regimen (Dumitru et al. 2021; Reynolds and Kollef 2021).

In this regard, new therapeutic strategies are urgent, especially for these microorganisms that are often resistant to antibiotics. Thus, the NEs were also evaluated against the bacteria K25-A2, K26-A2, K29-A2, and K31-A2, which are clinical isolates of *K. pneumoniae* and are efflux pump and biofilm producers from hospitals in Recife-PE, from the study by Scavuzzi et al. (2017)

In the present study, for these resistant clinical isolates, the NEs proved to be a promising strategy to potentiate the effects of this compound. It should be noted that these isolates are resistant to clavulanic acid-amoxicillin (AMC), amoxicillin (AMO), ATM, CAZ, cefoxitin (CFO), cefotaxime (CTX), CIP, LVX, nalidixic acid (NAL), norfloxacin (NOR), piperacillin/tazobactam (PIT), and trimethoprim/sulfamethoxazole (SUT). K29-A2 and K31-A2 strains are also resistant to ertapenem (ERT), IMP, and MEM. Therefore, drugs and other substances with the potential to combat them are of great relevance.

Therefore, the encapsulation of CUR in NEs is a promising therapeutic alternative, as it potentiates the antibacterial activity and enables administration by oral, nasal, transdermal, or intravenous routes for the treatment of intestinal, respiratory, skin, and systemic infections caused by *S. aureus*, *E. faecalis*, *A. baumannii*, *E. coli*, *K. pneumoniae*, and *P. aeruginosa*, as well as antibiotic-resistant strains of *K. pneumoniae*.

### Evaluation of the antibacterial activity of nanoemulsions in association with ceftazidime against clinical isolates of resistant *Klebsiella pneumoniae*

Intestinal and respiratory tract infections caused by antibiotic-resistant bacteria are among the ten leading causes of death and represent a challenge for public health and the world economy (Reynolds and Kollef 2021; Wang et al. 2020). *K. pneumoniae* is a gram-negative, capsulated bacterium found in the environment and is related to urinary and intestinal infections and pneumonia, in addition to catheter infections, especially in elderly patients with chronic diseases and patients with respiratory diseases or immunosuppressed (Dumitru et al. 2021).

In this regard, it is observed that the accumulation of several resistance mechanisms results in infections with high mortality rates due to the scarcity and inefficiency of therapies, requiring the development of new therapeutic options (Kofteridis et al. 2020; Nandhini et al. 2022).

Some antibiotics can be used for the treatment of patients affected by these microorganisms, including ceftazidime (CAZ) (Bassetti et al. 2019). These antibiotics have antibiofilm potential and are clinically used in therapy against bacterial infections caused by these bacteria (Hedayati et al. 2021). However, these drugs have high levels of toxicity, may have difficulty penetrating and reaching the innermost layers of the biofilm, and do not have versatile pharmaceutical forms for administration in different routes, such as, for example, oral, nasal, intravenous, and transdermal (Michelon et al. 2020).

In this sense, the use of combined antimicrobial therapy of nanoemulsions associated with a β-lactam, such as CAZ, for the treatment of infections caused by resistant strains is configured as a therapeutic alternative. In the present study, NE-2-CUR and NE-4-CUR were able to reduce the dose of ceftazidime against several resistant strains of *K. pneumoniae* that produce efflux pumps.

The NEs containing CUR were found to predominantly have a synergistic or additive profile with CAZ. Therefore, it is observed that the association of NEs with ceftazidime can be a promising strategy in the fight against diseases caused by these bacteria. In this sense, it is noteworthy that the mechanism of action of CAZ involves the binding of ceftazidime to penicillin-binding proteins (PBP) and results in cell lysis and death. This happens due to the inhibition of peptidoglycan synthesis, one of the structures responsible for the formation of the cell wall of bacteria (Schirmer et al. 2020).

In a similar manner, the antibacterial activity of phenolic compounds, such as those present in CUR, can be exhibited by two main mechanisms, through interaction with the cell membrane and cell wall of microorganisms, since CUR inhibits protein synthesis and damages the bacterial cell wall, causing cell death (Jiang et al. 2020). Furthermore, phenolic compounds interact with bacterial membrane proteins making hydrogen bonds with their hydroxyl groups, resulting in changes in membrane permeability, which lead to cell destruction. These compounds can also penetrate bacterial cells and coagulate cell contents, affecting the metabolism and causing cell destruction (Tian et al. 2009).

However, CUR has a broad-spectrum antibacterial property. CUR inhibit bacterial growth and bacterial virulence factors, blocking bacterial biofilm formation and preventing bacterial adhesion to host receptors through the bacterial quorum sensing regulation system (Abdulrahman et al. 2020). Zheng and co-workers (2020) confirm the antibacterial mechanism of CUR based on five targeting structures and two modes of action, including the inhibition of the bacterial QS system, effects on DNA, effects on proteins, damage to the cell wall and cell membrane, phototoxicity, and synergy. In this sense, the synergistic effect can possibly be explained by the combination of multiple mechanism of action of these substances.

Many studies have demonstrated the CUR antibacterial effect, using different indicators. Through synergistic effects, the dose of antibacterial activity can be reduced, and different components can act on microorganisms in different ways, thereby interfering with the mechanisms of pathogen resistance. CUR has synergy with various antibiotics or bacteriostatic agents, such as cephalosporin oxime, cefotaxime, vancomycin, tetracycline, ampicillin, evil Westwood, norfloxacin, ciprofloxacin, fish oil, and chitosan (Deepika et al. 2019).

Finally, CUR has different antibacterial effect on gram-positive bacteria and gram-negative bacteria, when comparing the synergistically use among other substances to CUR alone. When used alone, gram-positive bacteria are more sensitive to CUR, and gram-negative bacteria are more sensitive when they are used synergistically. However, the specific reason has not been elucidated yet (Liakopoulou et al. 2021).

### Evaluation of antibiofilm activity against resistant clinical isolates of *Klebsiella pneumoniae*

Biofilms are bacterial communities adhered to a surface, biotic or abiotic, surrounded by a polymeric extracellular matrix (Di Salle et al. 2021). This microbial community plays a protective role, ensuring the survival of microorganisms even in hostile environments (Blackman et al. 2021).

Approximately 80% of microbial infections are related to biofilm, so the biofilm plays a role as a reservoir of pathogens, causing serious infections, such as endocarditis, osteomyelitis, sinusitis, urinary tract infections, chronic prostatitis, periodontitis, otitis, and several other infections. Hospitals (Li et al. 2021; Srinivasan et al. 2021).

In this sense, it appears that the number of infections related to biofilm in hospital environments is significant (Nwabuife et al. 2022). In developed countries, approximately 80% of patients with cystic fibrosis develop a chronic infection in the lungs and paranasal sinuses. Additionally, of patients admitted to the ICU who use intravenous catheters, five out of 1000 develop catheter-related bloodstream infections, most caused by biofilm-producing microorganisms (Blackman et al. 2021).

Therefore, combating biofilm-producing bacteria is a problem, and products that have the potential to inhibit their formation are needed. The NEs produced in the present study inhibited the formation of biofilms in the evaluated clinical isolates. However, no activity of curcumin alone was observed. In this sense, it is observed that the encapsulation of this bioactive compound reverses this situation, probably due to the properties of CUR, which hinder the action of this compound (hydrophobicity, rapid degradation, photosensitivity, among others).

In addition, it appears that compounds that inhibit the formation of biofilms are important (Srinivasan et al. 2021), as they reduce the likelihood of developing resistance, and strategies are important to minimize dependence on antibiotics, preferably in the early stages of bacterial adherence, that is, before the biofilm is formed (Di Salle et al. 2021). The antibiofilm activity of curcumin, among other antibacterial properties, is related to its own oxidation. Curcumin autoxidizes at physiological pH and forms a spectrum of electrophilic and nucleophilic metabolites (peroxyl radical, endoperoxides, vinyl ether, cyclopentadiene, among others) (Gayani et al. 2019).

Also, CUR exerts an inhibitory effect on the biofilm formation process, reducing biomass, preventing adhesion, and destroying the biofilm structure through modulation of the QS system, greatly affecting bacterial resistance and thus reducing the use of bacteriostatic agents and the number of bacteria in the biofilm (Di Salle et al. 2021).

Therefore, CUR is a valuable therapeutic resource; however, new clinical studies must be carried out to promote therapeutic safety in its use against bacterial infections, and, although the eradication of biofilms was not observed with the NEs tested in the present study, the capacity of the nanotechnological products developed in this study to prevent the formation of the biofilm is a characteristic relevance, as biofilm-producing microorganisms contribute to the wide dissemination of multi-drug resistant strains, as these bacteria in the biofilm share resistance genes, contributing to the increase of these pathogens in the unit.

These bacteria are responsible for causing chronicity, persistence, and recurrence of infections, leading to high morbidity and mortality (Nwabuife et al. 2022). Therefore, substances that can inhibit the formation of biofilms are valuable, delaying the installation and persistence of pathologies caused by resistant and biofilm-forming bacteria.

It should be noted that biofilms are associated with antibacterial therapeutic failures, especially in infections related to health care (Blackman et al. 2021), causing longer hospitalization times and high morbidity and mortality rates, in addition to economic burden (Srinivasan et al. 2021). Thus, it becomes essential to discover substances that have antibacterial potential and can be used alone or combined with other substances in the treatment of infections, especially against bacteria that have resistance mechanisms.

Several in vitro studies have revealed the antimicrobial effects of curcumin against gram-negative and gram-positive bacteria and fungi. Different antimicrobial mechanisms have been demonstrated for curcumin, such as the inhibitory action on bacterial DNA replication, alteration of bacterial motility, and alteration of bacterial gene expression. Therefore, it is observed that curcumin interacts with various molecular targets and transduction pathways through multiple mechanisms (Abdulrahman et al. 2020).

Among the antibiofilm mechanisms, the decrease in extracellular polymeric substances (EPSs) stands out, which tends to reduce the amount of nutrients available for cell growth and surface conditions for greater adhesion; the inhibition of bacterial migration, by reducing the production of extracellular polysaccharides and alginate and the clustering of quorum sensing; the inhibition of virulence, since virulence factors can be regulated by signaling molecules of the quorum sensing system, for example, by reducing the expression levels of the *lasI* and *lasR* genes. The antibiofilm activity of curcumin is also related to its oxidation at physiological pH, forming electrophilic and nucleophilic metabolites (peroxyl radical, endoperoxides, vinyl ether, cyclopentadiene, among others) related to the cellular effects of curcumin (Di Salle et al. 2021).

Although the antibacterial activity of CUR is recognized, this free molecule did not show antibiofilm activity, probably due to the physical–chemical characteristics and properties of the substance (hydrophobicity, rapid degradation, and photosensitivity, among others). Therefore, once again the encapsulation of CUR in nanoemulsions overcomes this limitation of CUR, allowing this molecule to express its antibiofilm activity.

In this sense, the present research developed, characterized, and evaluated the antibacterial and antibiofilm activity of olive oil nanoemulsions containing curcumin from *Curcuma longa* L. (CUR). The spontaneous emulsification method followed by sonication proved to be effective in all preparations, and the chosen formulations presented ideal quality characteristics for in vivo testing, as well as for intradermal, oral, nasal, and intravenous administration.

NE-2-CUR and NE-4-CUR nanoemulsions were characterized and showed physicochemical stability for 120 days and in vitro antibacterial activity against several pathogenic bacteria. These NEs showed promise in terms of interaction with ceftazidime, with a predominantly additive or synergistic profile, reducing the necessary concentration of ceftazidime to eliminate clinical bacterial isolates of *K. pneumoniae*. Furthermore, NEs were able to inhibit the formation of biofilms in clinical isolates of *K. pneumoniae* that produce moderate and strong biofilms and are efflux pump producers.

Therefore, NE-2-CUR and NE-4-CUR alone or in combination therapy with ceftazidime are promising therapeutic alternatives for infections caused by several bacteria of medical importance.

Supplementary information.

## Supplementary Information

Below is the link to the electronic supplementary material.

Supplementary file1 (PDF 242 KB)

## Data Availability

The data concerning the clinical isolates of *Klebsiella pneumoniae* resistant to antibiotics and biofilm and efflux pump producers (K-25-A2, K-26-A2, K29-A2, and K-31-A2) that support the findings of this study are available in the National Center for Biotechnology Information at 10.1099/jmm.0.000452, reference Scavuzzi et al. (2017). Also, the authors confirm that the data supporting the findings of this study are available within the article and its supplementary materials. Finally, raw data were generated at iLIKA/UFPE (Recife, Pernambuco, Brazil) and at Central Analítica—USP Institute of Chemistry (São Paulo, São Paulo, Brazil), and derived data supporting the findings of this study are available from the corresponding author [MVAC] on request.
