# Supplementary material for: Olive oil nanoemulsion containing curcumin: antimicrobial agent against multidrug-resistant bacteria
Source: Appl Microbiol Biotechnol. 2024 Feb 27;108(1):241. doi: 10.1007/s00253-024-13057-x (PMC10899360; doi:10.1007/s00253-024-13057-x)

Applied Microbiology and Biotechnology

OLIVE OIL NANOEMULSION CONTAINING CURCUMIN: ANTIMICROBIAL AGENT AGAINST MULTIDRUG RESISTANT BACTERIA

Maine Virgínia Alves Confessor[1,2], Maria Anndressa Alves Agreles[1], Luís André de Almeida Campos[1], Azael Francisco Silva Neto[1], Joyce Cordeiro Borges[1], Rodrigo Molina Martins[2], Alexsandra Maria Lima Scavuzzi[3], Ana Catarina Souza Lopes[3], Elisangela Afonso de Moura Kretzschmar[4], Isabella Macário Ferro Cavalcanti[1,5]*

1 Federal University of Pernambuco (UFPE), Keizo Asami Institute (iLIKA), Recife, Pernambuco, Brazil
2 University Center UNIFACISA, Campina Grande, Paraiba, Brazil
3 Department of Tropical Medicine, Federal University of Pernambuco (UFPE), Recife, Pernambuco, Brazil
4 Federal University of Paraíba (UFPB), Department of Biotechnology, João Pessoa, Paraíba, Brazil
5 Federal University of Pernambuco (UFPE), Laboratory of Microbiology and Immunology, Academic Center of Vitória (CAV), Vitória de Santo Antão, Pernambuco, Brazil

*Corresponding author
Prof. Maine Virgínia Alves Confessor
Keizo Asami Institute (iLIKA), Federal University of Pernambuco (UFPE), Recife, Pernambuco, Brazil
Prof. Moraes Rego Avenue, 1235, Cidade Universitária, CEP: 50670-901, Fone: +55 (81) 21012501, Recife/PE, Brazil.
University Center UNIFACISA, Campina Grande, Paraiba, Brazil. Manoel Cardoso Palhano, 124-152 – Itararé, CEP: 58408-326, Fone: +55 (83) 2101-8877 Campina Grande, PB, Brazil.
E-mail: maine_alves@hotmail.com; maine.confessor@maaisunifacisa.com.br; maine.confessor@ebserh.gov.br

Figure S1: Supplementary data for TGA – NE-2.

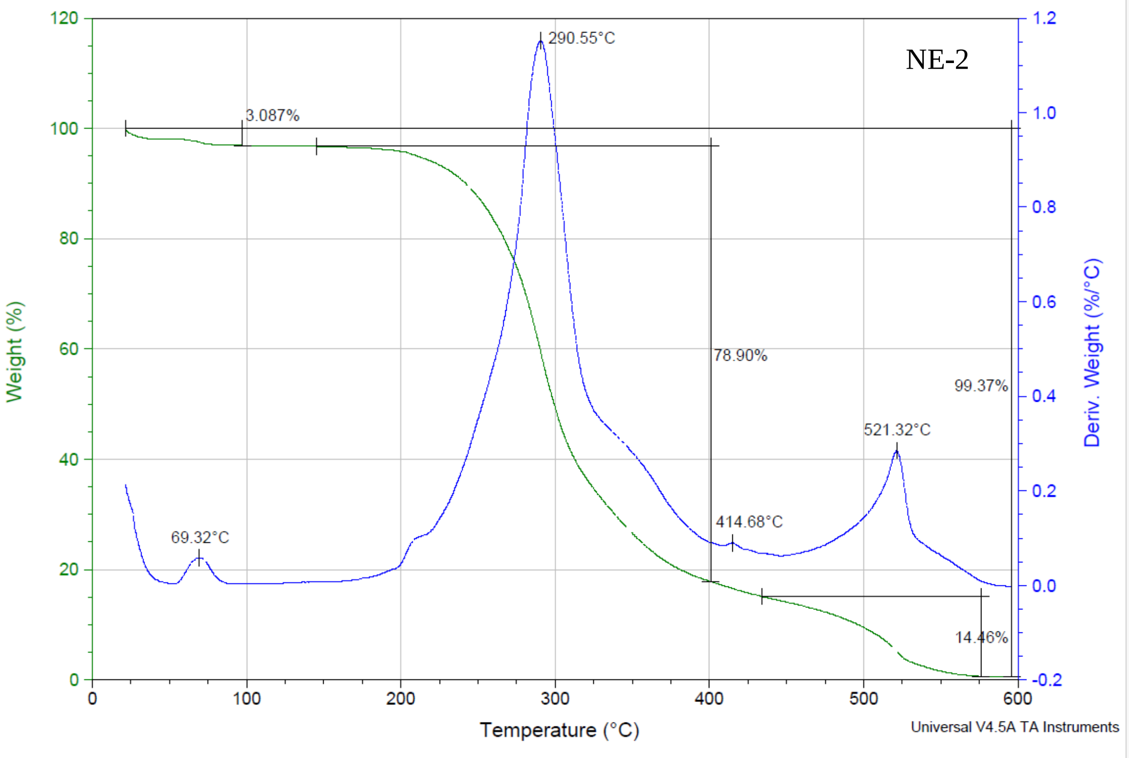

Figure S2: Supplementary data for TGA – NE-2-CUR.

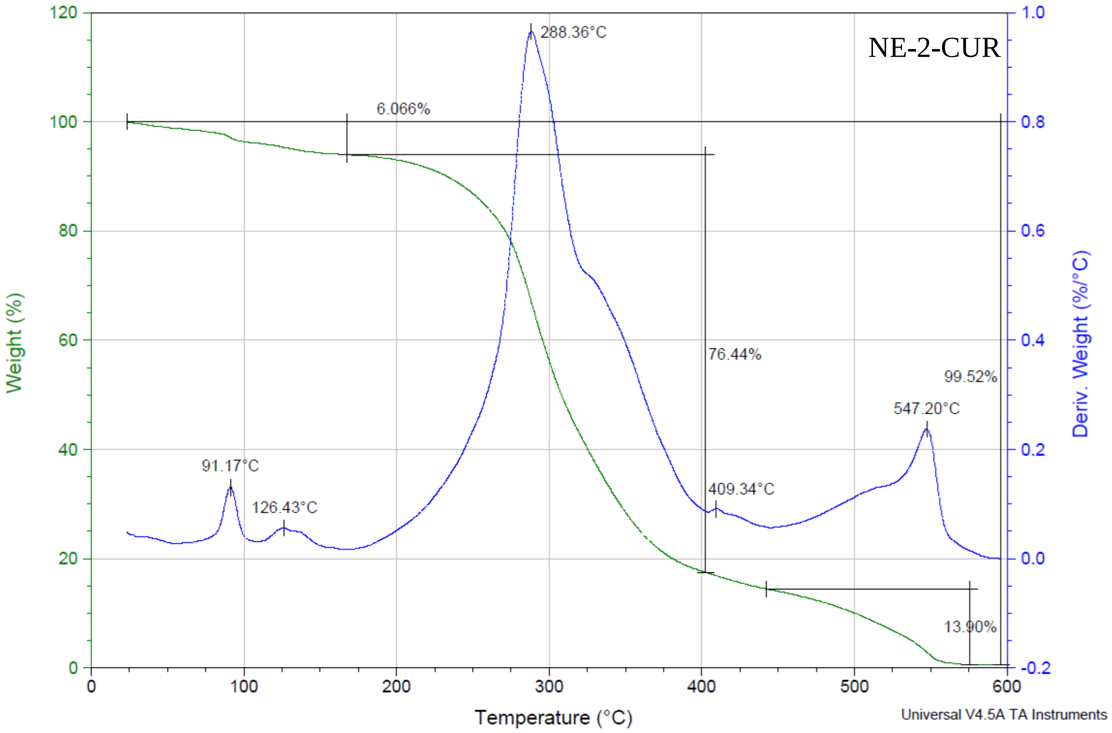

Figure S3: Supplementary data for TGA – NE-4-CUR.

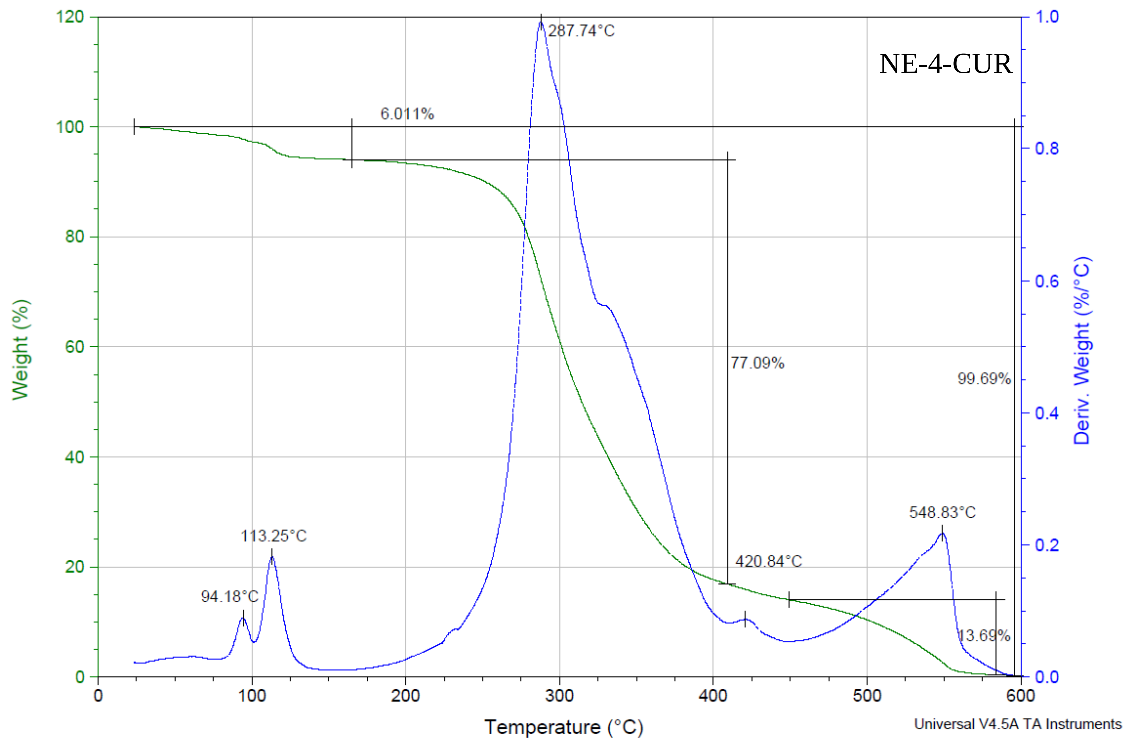

Figure S4: Supplementary data for TGA –CUR.

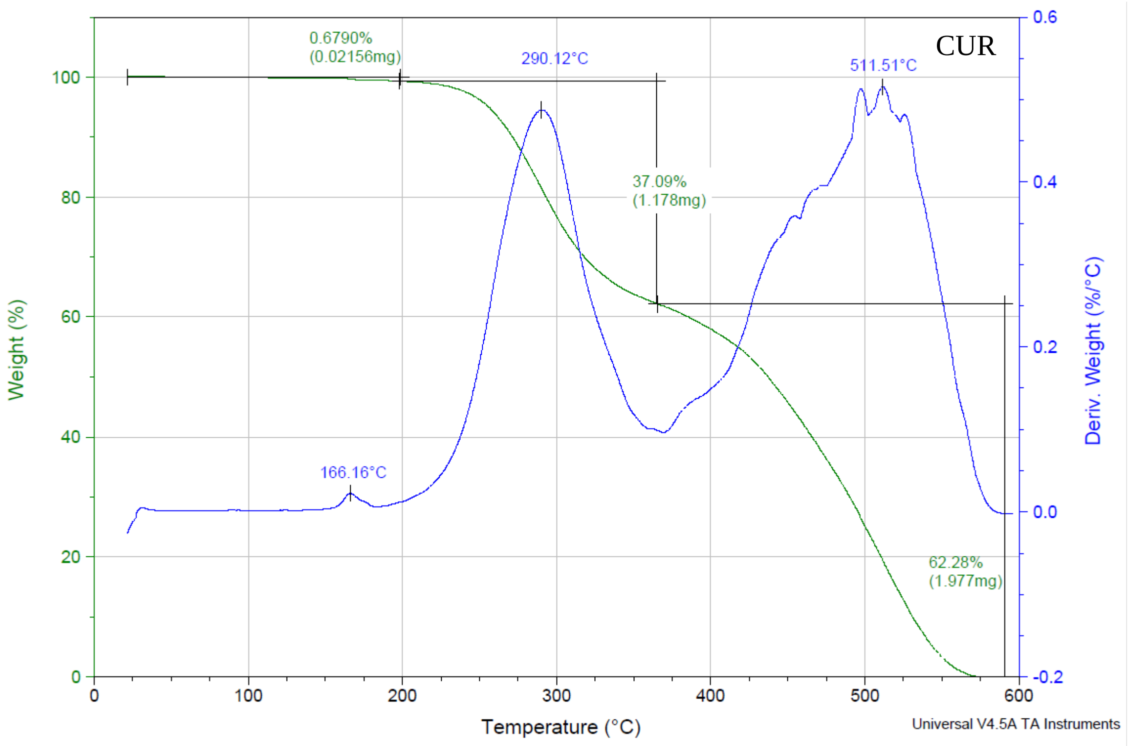

Supplement: Supplementary file 1 — Supplementary file1 (PDF 242 KB) [file 253_2024_13057_MOESM1_ESM.pdf]
